# Editorial for the Special Issue on High-Risk Localized and Locally Advanced Prostate Cancer

**DOI:** 10.3390/cancers15123153

**Published:** 2023-06-11

**Authors:** Kouji Izumi

**Affiliations:** Department of Integrative Cancer Therapy and Urology, Kanazawa University Graduate School of Medical Science, 13-1 Takara-machi, Kanazawa 920-8641, Ishikawa, Japan; azuizu2003@yahoo.co.jp

The recent development of imaging modalities, such as diffusion-weighted whole-body imaging with background suppression (DWIBS) and positron emission tomography of prostate-specific membrane antigen (PSMA-PET) with a radioactive diagnostic agent, has enabled the detection of minute metastases in patients diagnosed with high-risk localized and locally advanced prostate cancer by conventional modalities. The impact of imaging developments on prognosis has not been fully assessed. However, the increasing prevalence of cutting-edge imaging modalities may soon change the definitions of high-risk localized and locally advanced prostate cancer. Even now, around 20–30% of nonmetastatic prostate cancer patients have high-risk localized disease requiring curative treatment. The best treatment for high-risk localized disease is also still unclear, and the reliability of the definition of “high-risk” may need to be validated first. Although recent advances in radiotherapy for prostate cancer have yielded excellent long-term results, even in high-risk localized disease, there are no studies directly comparing it with local treatment options, such as prostatectomy. To address current challenges in high-risk localized and locally advanced prostate cancer, 12 original and 3 review articles covering aspects from basic research to clinical trials are published in this Special Issue.

Cancer aggressiveness

Ottman et al. investigated the factors involved in prostate cancer aggressiveness and racial disparity with miRNA expression studies and suggest a role for some miRNAs in prostate cancer diagnosis, prognosis, and the elimination of health disparities [1]. Diop et al. report that intraductal carcinoma of the prostate, an aggressive histological subtype, showed a reduced number of infiltrated immune cells compared to the surrounding tissues and it provided different survival [2]. Samarija et al. showed that amino acid metabolism-related gene expression is aberrant in prostate cancer. The expression of SERINC3 and CSAD genes strongly differentiated between better and worse prognosis for high and low Gleason scores, respectively [3]. Bui et al. examined the endogenous CCL2 functions in murine bone marrow-derived mesenchymal stem cells. They discovered that CCL2 plays a crucial role in prostate cancer growth within the tumor microenvironment [4]. Baba et al. reviewed 557 prostate cancer patients who underwent radical prostatectomy and found that a tumor volume over 2.8 cc was an independent predictive factor for biochemical recurrence. They also established a novel risk assessment model based on tumor volume and location [5].

Regional lymph node invasion

Di Pierro et al. evaluated the accuracy of the four most used nomograms for predicting lymph node invasion. Comparing them in high-risk prostate cancer patients, the predictive performance of the four nomograms was virtually the same, as was their ability to avoid unnecessary extended pelvic lymph node dissection. [6]. Yamashita et al. conducted a prospective study in high-risk prostate cancer patients. They aimed to assess the impact of lymphatic invasion on biochemical recurrence in patients who underwent robot-assisted radical prostatectomy and extended lymph node dissection. They found that lymphatic invasion in the primary site was a significant independent predictor of biochemical recurrence [7].

Radiotherapy

Faccenda et al. investigated the dosimetric impact on the target, and organs at risk, of intrafraction prostate motion and interfraction anatomical changes in dose-escalated linac-based stereotactic body radiation therapy [8]. Francolini et al. conducted the STARR trial and reported early toxicity and biochemical outcomes after stereotactic salvage radiotherapy for macroscopic recurrence within the prostate bed after radical prostatectomy. They demonstrated an optimal tolerability profile and promising oncologic outcomes [9]. Yamazaki et al. contributed to this Special Issue by providing two brachytherapy studies. They report a lower biochemical control rate and distant metastasis-free survival rate in patients with both T3b–4 and Gleason score 9–10, than in those with a single risk factor. They also evaluated the different role of brachytherapy boost according to each risk group [10,11]. Shih et al. compared intensity-modulated radiotherapy plus antiandrogen therapy and radical prostatectomy in relatively young patients (aged ≤ 65 years). Although both had similar oncological outcomes, radical prostatectomy showed a greater reduction in the risk of biochemical failure [12].

Reviews

Gogola et al. provide a synopsis of the transcription factors and signaling pathways involved in epithelial-to-non-epithelial (“mesenchymal”) transition prostate cancer progression [13]. Iwamoto et al. discuss the position, indications, complications, and prospects of androgen deprivation therapy for high-risk localized and locally advanced prostate cancer [14]. Finally, Makino et al. review the current literature with a focus on the definition of very high-risk prostate cancer, the role of modern imaging, and its treatment options [15].

I am very proud of this Special Issue; the papers are excellent, and I would like to thank all the authors. I would also like to acknowledge the reviewers for their time and careful appraisals of the manuscripts for this Special Issue. I believe that this publication will help physicians and academics involved in prostate cancer care and research, to better understand the essentials of “High-risk Localized and Locally Advanced Prostate Cancer.”
